# Concerns Expressed by Parents of Children with Pervasive Developmental Disorders for Different Time Periods of the Day: A Case–Control Study

**DOI:** 10.1371/journal.pone.0124692

**Published:** 2015-04-21

**Authors:** Yoshinori Sasaki, Masahide Usami, Daimei Sasayama, Takashi Okada, Yoshitaka Iwadare, Kyota Watanabe, Hirokage Ushijima, Tetsuya Tanaka, Maiko Harada, Hiromi Tanaka, Masaki Kodaira, Nobuhiro Sugiyama, Tetsuji Sawa, Kazuhiko Saito

**Affiliations:** 1 Department of Child and Adolescent Psychiatry, National Center for Global Health and Medicine, Kohnodai Hospital, Chiba, Japan; 2 Department of Psychiatry, Shinshu University, Matsumoto, Japan; 3 Department of Child and Adolescent Psychiatry, Nagoya University Graduate School of Medicine, Nagoya, Japan; 4 Department of Child and Adolescent Mental Health, Imperial Gift Foundation, Aiiku Maternal and Child Health Center, Aiiku Hospital, Tokyo, Japan; 5 Department of Developmental Psychiatry, Graduate School of Medical Science, Kitasato University, Tokyo, Japan; TNO, NETHERLANDS

## Abstract

**Background/Aim:**

The Questionnaire: Children with Difficulties (QCD) is a parent-assessed questionnaire designed to evaluate child’s difficulties in functioning during specific periods of the day. This study aimed to evaluate difficulties in daily functioning of children and adolescents with pervasive developmental disorder (PDD) using the QCD. Results were compared with those for a community sample.

**Methods:**

A case–control design was used. The cases comprised elementary school students (182 males, 51 females) and junior high school students (100 males, 39 females) with PDD, whereas a community sample of elementary school students (568 males, 579 females) and junior high school students (180 males, 183 females) was enrolled as controls. Their behavior was assessed using the QCD, the Tokyo Autistic Behavior Scale (TABS), the ADHD-rating scale (ADHD-RS), and the Oppositional Defiant Behavior Inventory (ODBI) for elementary and junior high school students, respectively. Effects of gender and diagnosis on the QCD scores were analyzed. Correlation coefficients between QCD and TABS, ADHD-RS, and ODBI scores were analyzed.

**Results:**

The QCD scores for the children with PDD were significantly lower compared with those from the community sample (P < 0.001). Significantly strong correlations were observed in more areas of the ADHD-RS and ODBI scores compared with the TABS scores.

**Conclusions:**

Children with PDD experienced greater difficulties in completing basic daily activities; moreover, their QCD scores revealed stronger associations with their ADHD-RS and ODBI scores in comparison with their TABS scores. The difficulties of PDD, ADHD and OBDI symptoms combined in children makes it necessary to assess all diagnoses before any therapy for PDD is initiated in order to be able to evaluate its results properly.

## Introduction

Pervasive developmental disorder (PDD), one of the most common developmental disorders, is a neurodevelopmental disorder presenting with persistent core symptoms of qualitative and quantitative failure of communication and delay in language development [1]. It has been suggested that children with PDD experience difficulties in multiple domains, including family relationships, school life, and friendships and that their families have to bear a significant burden [2,3]. Teachers reported that males with PDD had greater externalizing and social problems than females [4].

Symptoms of attention deficit hyperactivity disorder (ADHD) such as hyperactivity, impulsiveness, and inattention are frequent among individuals with PDD [2–7]. The diagnostic criteria for Autism Spectrum Disorder in the Diagnostic and Statistical Manual of Mental Disorders, 5^th^ edition (DSM-5), include ADHD for comorbid disorders. Furthermore, ADHD is often comorbid with other neuropsychiatric disorders [6], of which the oppositional defiant disorder (ODD) is common. Children with severe ADHD often had ODD for comorbid disorder [8]. Therefore, we examined both ADHD and ODD symptoms in children with PDD in this study.

The application of pharmacotherapy in ADHD is determined thorough a detailed assessment of symptoms and developmental levels in the child [9]. In Japan, only two drugs are currently approved for the management of ADHD, the long-acting methylphenidate and the nonstimulant atomoxetine [10]. In fact, methylphenidate or atomoxetine can be clinically useful for managing ADHD symptoms in children with autism [11–13]. When determining the need for pharmacotherapy in PDD, it is very important to assess ADHD symptoms during different time periods of the day to ensure that symptoms are pervasive. Furthermore, several challenges exist in the evaluation of children with PDD. To date, we are unaware of any study evaluating problems experienced during different time periods of the day by children with PDD.

ADHD-RS, a Japanese version of the ADHD-Rating Scale-IV published in 2008 [14,15], is widely used for the evaluation of ADHD symptoms in Japan. However, ADHD-RS only considers core symptoms of ADHD, and does not assess difficulties associated with daily functioning. Concurrently, the Child Behavior Checklist (CBCL) was introduced in Japan and has established reliability and validity [16]. Because of the large number of parameters, CBCL is inconvenient for use in daily practice and is unsuitable for repeated evaluation throughout the day. An alternative tool is the Questionnaire: Children with Difficulties (QCD) constructed by Yamashita [17], which is a parent-assessed questionnaire designed to evaluate a child’s difficulties in functioning during specific time periods of the day. Because of the small number of parameters, the QCD is convenient for use in clinical practice.

Using the QCD, this study aimed to evaluate difficulties in daily functioning of children and adolescents with PDD. The primary hypothesis was that the QCD scores of children with PDD would significantly correlate with PDD, ADHD and ODD symptoms at all time periods of the day. In addition, we had two minor hypotheses. First, we hypothesized that the QCD scores of children with PDD would more strongly correlate with PDD symptoms than ADHD and ODD symptoms during all time periods of the day. Second the QCD scores of males with PDD would be significantly lower compared with those of females. These hypotheses indicated that when clinicians want to evaluate the difficulties in daily life of children with PDD, they should' not only evaluate PDD symptoms but also ADHD and ODD symptoms in these children.

## Methods

### Study Design and Setting

A retrospective case—control design was used to evaluate the daily life of children and adolescents with PDD using the QCD. Participants were divided into a PDD group (cases) and a community group (controls) for elementary and junior high school students, respectively. Groups comprised elementary and junior high school students from Ichikawa City, which is situated in the western part of Chiba Prefecture, facing Tokyo, across the Edogawa River. Located approximately 20 km from Tokyo’s metropolitan area, the city has fully developed into a residential area and a center of education. The population is estimated at 471,104 (as of April 2008), the fourth largest in the prefecture.

Our investigation is conducted according to the principles expressed in the Declaration of Helsinki.

Informed consent was received from the subjects in accordance to the "Ethical Guidelines for Clinical Epidemiology Research" Ministry of Health, Labour and Welfare. The guidelines state "It is not always necessary to obtain informed consent from study participants. However, researchers must publish information on the implementation of the study, including the purpose of the study" for observational studies only using past clinical records and not human tissue samples.

The study’s purpose, method, inquiry, and how to refuse participation was posted in the hospital’s outpatient clinic. In addition, verbal consent was obtained from the subjects, and the dates used in this study were anonymized because date-patient correspondence was unnecessary throughout the study.

The ethical committee of the National Center for Global Health and Medicine approved this consent procedure of this study in reference to both the PDD group and the control community group.

The ethical committee of the National Center for Global Health and Medicine approved this retrospective study.

### Recruitment and Participants

#### Cases (PDD group)

All individuals in the PDD group were examined and diagnosed with PDD according to the Diagnostic and Statistical Manual of Mental Disorders, Fourth Edition, Text Revision, (DSM-IV-TR) [1]. All diagnoses were made by psychiatrists specializing in child and adolescent psychiatry at the Department of Child and Adolescent Psychiatry, Kohnodai Hospital, National Center for Global health and Medicine, between September 4, 2008 and May 11, 2012. Data from four questionnaires, QCD, ADHD-RS, the Tokyo Autistic Behavior Scale (TABS) [18], and the Oppositional Defiant Behavior Inventory (ODBI) [19], were obtained from patients’ clinical records. Students with other coexisting mental disorders, including mental retardation, ODD, or conduct disorder, were excluded in the results by reference to such psychological tests and questionnaires, psychiatrists specializing in child and adolescent psychiatry interviewed the parents and students. The dates were anonymized at that point. Clinical data, such as name and birth date were anonymized. We only use the clinical data such as age and the results of questionnaires without personal information.

#### Controls (community sample)

We explained the study outline to members of the educational committee in Ichikawa City and obtained their approval to perform a survey of public elementary and junior high school students. A consent form, an assent form, and four questionnaires (QCD, ADHD-RS, TABS, and ODBI) were distributed by teachers to parents of 10,000 randomly selected children between September 20, 2008 and September 30, 2008. The dates were anonymized at that point. Data, such as name and birth date were anonymized. We only use the data such as age and the results of questionnaires without personal information.

### Measures

#### QCD (Questionnaire-Children with Difficulties)

QCD comprises 20 questions related to activities during specific time periods of the day: questions 1–4, early morning and before going to school; 5–7, during school; 8–10, after school; 11–14, during the evening; 15–18, at night; and, 19–20, overall behavior (See S1 Appendix). Each question is scored as follows: 0 = completely disagree; 1 = somewhat (partially) agree; 2 = mostly agree; and 3 = completely agree. Higher scores indicate higher life function and less difficulty. Questions are designed to be practical and easy-to-understand such as washing one’s face, brushing one’s teeth, and getting dressed. For evaluating reliability, Cronbach’s alpha was calculated to assess the internal consistency of the questionnaire. The internal consistency and validity of the QCD have previously been demonstrated [20].

#### ADHD-RS (ADHD-Rating Scale)

This is an 18-item measure of ADHD symptoms used with children [13]. Tanaka et al. standardized the Japanese version of ADHD-RS, which has two factors: ‘‘hyperactivity and impulsiveness’” and “‘inattention”‘. There are four possible responses per question, and higher scores indicate multiple and more severe symptoms.

#### Tokyo Autistic Behavior Scale (TABS)

This tool comprises 39 items that are provisionally grouped in four areas: interpersonal—social relationship, language—communication, habit—mannerism, and others. It is used by a child’s caretaker to rate the child’s autistic behaviors on a three-point scale. Higher scores indicate multiple and more severe symptoms [18].

#### Oppositional Defiant Behavior Inventory (ODBI)

This comprised 41 questions, covering DSM-IV-TR diagnostic criteria for ADHD, ODD, and conduct disorder (CD). Items are worded as closely as possible to DSM-IV-TR, using a rating scale format. Each item is rated on a four-point scale from “0” (not at all) to “3” (very much). Subjects with ODBI scores over 20 were considered to have ODD (a high ODBI subgroup). Internal consistency, test—retest reliability, concurrent validity, and divergent validity of ODBI have previously been examined [19].

### Statistical analysis

Scores for questions in each of the six subcategories and total QCD scores were separately determined and were statistically compared between the PDD group and the community sample using an unpaired t-test, and effect sizes were calculated.

Effects of gender and diagnosis (PDD group vs. community sample) on total QCD scores were analyzed using two-way analysis of variance (ANOVA). We considered the interaction to examine whether there were differences in the QCD scores by combined factors such as gender, diagnosis. Spearman’s correlation coefficient was calculated to determine whether total QCD scores and subscores correlated with TABS, ADHD-RS, and ODBI scores.

All statistical tests were two-tailed, and the significance threshold was defined as P < 0.05. The absolute value of the correlation coefficient was regarded as a significantly strong correlation of 0.4 or more. Analyses were performed using the PASW Statistics 18.0 statistical software (IBM Japan Incorporated). Raw data used for analyses described in this manuscript are available upon request.

## Results

### Participants and descriptive data

The PDD group comprised 233 elementary and 139 junior high school students diagnosed with PDD according to DSM-IV-TR and the community sample group comprised 1,147 public elementary and 363 public junior high school students.

#### Cases (PDD group)

In elementary school, 233 individuals (182 males, 51 females), with an average age of 8.76 ± 1.83 years (mean ± standard deviation; range, 6–12 years) were included in the PDD group. Among the junior high school students, 139 individuals (100 males, 39 females) with an average age 13.3 ± 1.00 years (mean ± standard deviation; range, 12–15 years) were included in the PDD group.

#### Controls (community sample)

Questionnaires were retrieved from 1,802 parents who provided informed consent to the mailed survey. Of these, 1,510 questionnaires that were completely filled were analyzed. Therefore, the community sample group comprised 1,147 public elementary school students (568 males, 579 females) and 363 junior high school students (180 males, 183 females). The average age of participants in public elementary students was 8.63 ± 1.75 years (range, 6–12 years). The average age of participants in public junior high school students was 13.1 ± 0.92 years (range, 12–15 years).

The age difference between the cases (8.76 ± 1.83) and controls (8.63 ± 1.75) for elementary school students was not significantly. However, the age difference between the cases (13.3 ± 1.00) and controls (13.1 ± 0.92) for junior high school students was significantly(p<0.05 and effect size 0.21).

### Outcome data

#### Distributions of the QCD Scores

QCDs that were completely filled were collected from parents of children from both the PDD group and community sample (Tables 1 and 2). All six QCD subscores and total scores were significantly lower in the PDD group than in the community sample (P < 0.001) for both elementary and junior high school students. An effect size of “overall behavior” was highest for elementary school students in all QCD subscores. Similarly an effect size during “after school” was highest for junior high school students in all QCD subscores.

**Table 1:** Clinical data for the PDD group and the community sample (elementary school).

		PDD group	Community sample	P value	Effect size
**Number (boy/girl)**		233(182/51)	1147(568/579)		
**Age (mean ± SD)**		8.76±1.83	8.63±1.75	NS	0.07
**QCD score (mean ± SD)**	Morning	4.78±2.99	7.92±2.80	<0.001	1.11
	School	4.73±2.20	7.78±1.54	<0.001	1.83
	After school	4.18±2.31	7.45±1.69	<0.001	1.80
	Evening	5.80±3.06	9.66±2.25	<0.001	1.61
	Night	6.40±2.29	7.95±1.47	<0.001	0.95
	Overall behavior	2.11±1.55	4.72±1.33	<0.001	1.91
	Total score	28.0±10.3	45.5±8.35	<0.001	2.01
**TABS score (mean ± SD)**	Total score	14.8±7.46	5.26±4.50	<0.001	1.87
**ADHD-RS (mean ± SD)**	Total score	23.7±13.0	5.46±6.73	<0.001	2.25
**ODBI (mean ± SD)**	Total score	27.0±13.4	13.5±10.1	<0.001	1.26

**Score ranges**

QCD (Questionnaire-Children with Difficulties): Morning 0~12points, School 0~9points, After school 0~9points, Evening 0~12points, Night 0~12points, Overall behavior 0~6points, Total score 0~60points.

TABS (Tokyo Autistic Behavior Scale): 0~39points

ADHD-RS (ADHD-Rating Scale): 0~54points

ODBI (Oppositional Defiant Behavior Inventory): 0~54points

**Table 2:** Clinical data for the PDD group and the community sample (junior high school).

		PDD group	Community sample	P value	Effect size
**Number (boy/girl)**		139(100/39)	363(180/183)		
**Age (mean ± SD)**		13.3±1.00	13.1±0.92	<0.05	0.21
**QCD score (mean ± SD)**	Morning	5.42±3.25	8.58±2.66	<0.001	1.11
	School	4.37±2.31	7.60±1.69	<0.001	1.72
	After school	3.91±2.27	7.47±1.70	<0.001	1.90
	Evening	6.49±3.12	9.50±2.41	<0.001	1.15
	Night	5.96±2.60	8.04±1.68	<0.001	1.05
	Overall behavior	2.14±1.55	4.74±1.38	<0.001	1.83
	Total score	28.3±10.4	45.9±8.82	<0.001	1.90
**TABS score (mean ± SD)**	Total score	11.0±6.58	4.68±4.24	<0.001	1.27
**ADHD-RS (mean ± SD)**	Total score	17.7±11.1	4.32±6.13	<0.001	1.72
**ODBI (mean ± SD)**	Total score	21.0±13.6	11.5±9.14	<0.001	0.90

**Score ranges**

QCD (Questionnaire-Children with Difficulties): Morning 0~12points, School 0~9points, After school 0~9points, Evening 0~12points, Night 0~12points, Overall behavior 0~6points, Total score 0~60points.

TABS (Tokyo Autistic Behavior Scale): 0~39points

ADHD-RS (ADHD-Rating Scale): 0~54points

ODBI (Oppositional Defiant Behavior Inventory): 0~54points

#### QCD Scores by Gender in the PDD group and QCD Scores by Diagnosis

Total QCD scores and six QCD subscores of children in the PDD group were compared on the basis of the gender of participants (Tables 3 and 4). The total QCD score, “School” and “after school” subscores were significantly lower for males than that for female for elementary school students in the PDD group (P < 0.05). Moreover, the average total QCD score and QCD subscores were compared on the basis of diagnosis (Tables 5 and 6). The total scores and subscores were significantly lower in the PDD group than that in the community sample when compared on the basis of diagnosis for both elementary school students and junior high school students (P < 0.01). There were no interactions made by combined factors such as gender, diagnosis. For elementary school students, “Morning” subscore was significantly lower(F(1,1376) = 157.2,p<0.01), “School” subscore was significantly lower(F(1,1376) = 413.7,p<0.01), “After school” subscore was significantly lower(F(1,1376) = 383.6,p<0.01), “Evening” subscore was significantly lower(F(1,1376) = 320.9,p<0.01), “Night” subscore was significantly lower(F(1,1376) = 117.6,p<0.01), “Overall behavior” subscore was significantly lower(F(1,1376) = 464.0,p<0.01), Total score was significantly lower(F(1,1376) = 504.2,p<0.01) on the basis of diagnosis. For junior high school students, “Morning” subscore was significantly lower(F(1,498) = 104.1,p<0.01), “School” subscore was significantly lower(F(1,498) = 247.4,p<0.01), “After school” subscore was significantly lower(F(1,498) = 287.8,p<0.01), “Evening” subscore was significantly lower(F(1,498) = 114.2,p<0.01), “Night” subscore was significantly lower(F(1,498) = 93.7,p<0.01), “Overall behavior” subscore was significantly lower(F(1,498) = 300.9,p<0.01), Total score was significantly lower(F(1,498) = 306.3,p<0.01) on the basis of diagnosis.

**Table 3:** QCD Scores of children by gender in the PDD group (elementary school).

		Boys	Girls	P value	Effect size
**Number**		182	51		
**Age (mean ± SD)**		8.78±1.84	8.66±1.80	NS	0.07
**QCD score (mean ± SD)**	Morning	4.67±2.98	5.16±2.97	NS	0.17
	School	4.57±2.19	5.33±2.12	<0.05	0.35
	After school	3.93±2.27	5.08±2.24	<0.01	0.51
	Evening	5.60±3.07	6.49±2.92	NS	0.29
	Night	6.36±2.32	6.53±2.19	NS	0.07
	Overall behavior	2.01±1.54	2.47±1.55	NS	0.30
	Total score	27.1±10.2	31.1±10.3	<0.05	0.39

**Score ranges**

QCD (Questionnaire-Children with Difficulties): Morning 0~12points, School 0~9points, After school 0~9points, Evening 0~12points, Night 0~12points, Overall behavior 0~6points, Total score 0~60points.

**Table 4:** QCD Scores of children by gender in the PDD group (junior high school).

		Boys	Girls	P value	Effect size
**Number**		100	39		
**Age (mean ± SD)**		13.4±0.98	13.3±1.08	NS	0.04
**QCD score (mean ± SD)**	Morning	5.38±2.94	5.54±3.98	NS	0.05
	School	4.30±2.44	4.54±1.94	NS	0.10
	After school	3.73±2.23	4.36±2.35	NS	0.29
	Evening	6.49±3.06	6.49±3.31	NS	0.00
	Night	5.96±2.58	5.97±2.68	NS	0.06
	Overall behavior	2.22±1.52	1.92±1.61	NS	0.20
	Total score	28.1±10.1	28.8±11.4	NS	0.07

**Score ranges**

QCD (Questionnaire-Children with Difficulties): Morning 0~12points, School 0~9points, After school 0~9points, Evening 0~12points, Night 0~12points, Overall behavior 0~6points, Total score 0~60points.

**Table 5:** Total QCD score and subscores (elementary school).

		PDD group			Community sample					
		M	SD	N	M	SD	N		F	p
**Morning**	Boys	4.67	2.98	182	7.76	2.83	568	Gender×Diag	0.13	NS
**(Q1∼Q4)**	Girls	5.16	2.97	51	8.07	2.76	579	Diag	157.2	< 0.01
								Gender	2.78	NS
**School**	Boys	4.57	2.19	182	7.52	1.63	568	Gender×Diag	0.81	NS
**(Q5∼Q7)**	Girls	5.33	2.12	51	8.04	1.39	579	Diag	413.7	< 0.01
								Gender	21.3	< 0.01
**After school**	Boys	3.93	2.27	182	7.12	1.83	568	Gender×Diag	2.71	NS
**(Q8∼Q10)**	Girls	5.08	2.24	51	7.77	1.47	579	Diag	383.6	< 0.01
								Gender	35.8	< 0.01
**Evening**	Boys	5.60	3.07	182	9.31	2.37	568	Gender×Diag	0.25	NS
**(Q11∼Q14)**	Girls	6.49	2.92	51	10.0	2.07	579	Diag	320.9	< 0.01
								Gender	15.2	< 0.01
**Night**	Boys	6.36	2.32	182	7.91	1.49	568	Gender×Diag	0.12	NS
**(Q15∼Q18)**	Girls	6.53	2.19	51	7.98	1.46	579	Diag	117.6	< 0.01
								Gender	0.75	NS
**Overall behavior**	Boys	2.01	1.54	182	4.56	1.40	568	Gender×Diag	0.41	NS
**(Q19∼Q20)**	Girls	2.47	1.55	51	4.87	1.24	579	Diag	464.0	< 0.01
								Gender	11.6	< 0.01
**Total score**	Boys	27.1	10.2	182	44.2	8.89	568	Gender×Diag	0.87	NS
	Girls	31.1	10.3	51	46.7	7.56	579	Diag	504.2	< 0.01
								Gender	19.7	< 0.01

df (1,1376)

**Score ranges**

QCD (Questionnaire-Children with Difficulties): Morning 0~12points, School 0~9points, After school 0~9points, Evening 0~12points, Night 0~12points, Overall behavior 0~6points, Total score 0~60points.

**Table 6:** Total QCD score and subscores (junior high school).

		PDD group			Community sample					
		M	SD	N	M	SD	N		F	p
**Morning**	Boys	5.38	2.94	100	8.27	2.69	180	Gender×Diag	0.53	NS
**(Q1∼Q4)**	Girls	5.54	3.98	39	8.87	2.58	183	Diag	104.1	< 0.01
								Gender	1.55	NS
**School**	Boys	4.30	2.44	100	7.37	1.78	180	Gender×Diag	0.31	NS
**(Q5∼Q7)**	Girls	4.54	1.94	39	7.83	1.55	183	Diag	247.4	< 0.01
								Gender	3.02	NS
**After school**	Boys	3.73	2.23	100	7.22	1.78	180	Gender×Diag	0.13	NS
**(Q8∼Q10)**	Girls	4.36	2.35	39	7.70	1.58	183	Diag	287.8	< 0.01
								Gender	7.61	< 0.01
**Evening**	Boys	6.49	3.06	100	9.13	2.51	180	Gender×Diag	1.77	NS
**(Q11∼Q14)**	Girls	6.49	3.31	39	9.87	2.23	183	Diag	114.2	< 0.01
								Gender	1.74	NS
**Night**	Boys	5.96	2.58	100	8.00	1.67	180	Gender×Diag	0.02	NS
**(Q15∼Q18)**	Girls	5.97	2.68	39	8.08	1.70	183	Diag	93.7	< 0.01
								Gender	0.05	NS
**Overall behavior**	Boys	2.22	1.52	100	4.61	1.47	180	Gender×Diag	3.19	NS
**(Q19∼Q20)**	Girls	1.92	1.61	39	4.86	1.26	183	Diag	300.9	< 0.01
								Gender	0.02	NS
**Total score**	Boys	28.1	10.1	100	44.6	9.12	180	Gender×Diag	0.90	NS
	Girls	28.8	11.4	39	47.2	8.28	183	Diag	306.3	< 0.01
								Gender	2.85	NS

df (1,498)

**Score ranges**

QCD (Questionnaire-Children with Difficulties): Morning 0~12points, School 0~9points, After school 0~9points, Evening 0~12points, Night 0~12points, Overall behavior 0~6points, Total score 0~60points.

#### Correlation with TABS, ADHD-RS, and ODBI

Correlations of total QCD scores and six QCD subscores with total TABS, ADHD-RS, and ODBI scores are presented in Tables 7 and 8. All correlations were significant. Significantly strong correlations were observed between the “evening” and “night” subscores with total TABS scores (ρ > −0.42, P < 0.05) for females with PDD for elementary school students. Significantly strong correlations were observed between “evening” subscores and total QCD scores with total TABS scores (ρ > −0.46, P < 0.05) for females with PDD for junior high school students. Significantly strong correlations were observed between total QCD scores and “evening” subscores with total ADHD-RS scores (ρ > −0.46, P < 0.05) for all children with PDD for elementary school students. Significantly strong correlations were observed between total QCD scores and “morning” subscores with total ADHD-RS scores (ρ > −0.43, P < 0.05) for females and between “evening” subscores with total ADHD-RS scores (ρ > −0.48, P < 0.05) for all children with PDD for junior high school students. Significantly strong correlations were observed between “morning” subscores with total ODBI scores (ρ > −0.40, P < 0.05) for males and between total QCD scores, “evening” and “overall behavior” subscores with total ODBI scores (ρ > −0.45, P < 0.05) for all children with PDD for elementary school students. Furthermore, significantly strong correlations were observed between “morning” subscores with total ODBI scores (ρ > −0.40, P < 0.05) for females and between total QCD scores, “evening” and “overall behavior” subscores with total ODBI scores (ρ > −0.44, P < 0.05) for all children with PDD for junior high school students.

**Table 7:** Correlation of the QCD score with the TABS, ADHD-RS and ODBI scores (elementary school).

		TABS		ADHD-RS		ODBI	
		PDD group	Community sample	PDD group	Community sample	PDD group	Community sample
**Morning**	Boys	-0.170*	-0.309*	-0.316*	**-0.404** *	**-0.406** *	**-0.403** *
**(Q1∼Q4)**	Girls	-0.234	-0.283*	-0.294*	**-0.464** *	-0.359*	-0.375*
**School**	Boys	-0.184*	-0.358*	-0.262*	**-0.418** *	-0.207*	-0.284*
**(Q5∼Q7)**	Girls	-0.248	-0.202*	-0.310*	-0.240*	-0.199	-0.173*
**After school**	Boys	-0.368*	-0.387*	-0.328*	**-0.412** *	-0.135	-0.295*
**(Q8∼Q10)**	Girls	-0.279*	-0.291*	-0.361*	-0.314*	-0.112	-0.190*
**Evening**	Boys	-0.332*	**-0.414** *	**-0.475** *	**-0.559** *	**-0.540** *	**-0.546** *
**(Q11∼Q14)**	Girls	**-0.486** *	-0.395*	**-0.490** *	**-0.523** *	**-0.572** *	**-0.524** *
**Night**	Boys	-0.220*	-0.290*	-0.227*	-0.280*	-0.330*	-0.267*
**(Q15∼Q18)**	Girls	**-0.421** *	-0.289*	-0.319*	-0.327*	-0.263	-0.247*
**Overall behavior**	Boys	-0.268*	**-0.427** *	-0.276*	**-0.500** *	**-0.469** *	**-0.538** *
**(Q19∼Q20)**	Girls	-0.244	-0.333*	-0.353*	**-0.421** *	**-0.553** *	**-0.448** *
**Total score**	Boys	-0.355*	**-0.472** *	**-0.464** *	**-0.573** *	**-0.490** *	**-0.513** *
	Girls	-0.396*	**-0.424** *	**-0.490** *	**-0.567** *	**-0.455** *	**-0.475** *

Bold: correlation coefficient< −0.40

* p<.05

QCD (Questionnaire-Children with Difficulties)

TABS (Tokyo Autistic Behavior Scale)

ADHD-RS (ADHD-Rating Scale)

ODBI (Oppositional Defiant Behavior Inventory)

**Table 8:** Correlation of the QCD score with the TABS, ADHD-RS and ODBI scores (junior high school).

		TABS		ADHD-RS		ODBI	
		PDD group	Community sample	PDD group	Community sample	PDD group	Community sample
**Morning**	Boys	-0.083	-0.328*	-0.335*	-0.370*	-0.392*	**-0.428** *
**(Q1∼Q4)**	Girls	-0.202	-0.295*	**-0.434** *	**-0.528** *	**-0.408** *	**-0.531** *
**School**	Boys	-0.077	-0.358*	-0.188	-0.396*	-0.102	-0.284*
**(Q5∼Q7)**	Girls	-0.240	-0.185*	-0.053	-0.224*	-0.082	-0.204*
**After school**	Boys	-0.242*	**-0.420** *	-0.101	**-0.420** *	-0.092	-0.240*
**(Q8∼Q10)**	Girls	-0.367*	-0.303*	-0.209	-0.387*	-0.148	-0.241*
**Evening**	Boys	-0.257*	-0.319*	**-0.489** *	**-0.548** *	**-0.534** *	**-0.590** *
**(Q11∼Q14)**	Girls	**-0.508** *	**-0.444** *	**-0.704** *	**-0.516** *	**-0.598** *	**-0.601** *
**Night**	Boys	-0.185	-0.321*	-0.118	-0.305*	-0.166	-0.298*
**(Q15∼Q18)**	Girls	-0.200	-0.289*	-0.200	-0.214*	-0.149	-0.204*
**Overall behavior**	Boys	-0.158	**-0.476** *	-0.253*	**-0.500** *	**-0.600** *	**-0.540** *
**(Q19∼Q20)**	Girls	-0.390*	**-0.552** *	-0.349*	**-0.490** *	**-0.570** *	**-0.495** *
**Total score**	Boys	-0.248*	**-0.483** *	-0.379*	**-0.566** *	**-0.449** *	**-0.552** *
	Girls	**-0.468** *	**-0.450** *	**-0.523** *	**-0.561** *	**-0.468** *	**-0.543** *

Bold: correlation coefficient< −0.40

* p<.05

QCD (Questionnaire-Children with Difficulties)

TABS (Tokyo Autistic Behavior Scale)

ADHD-RS (ADHD-Rating Scale)

ODBI (Oppositional Defiant Behavior Inventory)

## Discussion

To the best of our knowledge, this may well be the first study to examine the QCD scores in relation to TABS, ADHD-RS and OBDI scores during different time periods of the day between a community sample and children diagnosed with PDD. We observed that the QCD scores were significantly lower for the children with PDD than those for the community sample at each time period for both elementary and junior high school students. As we mentioned in the paragraph of Outcome data, the primary hypothesis that the QCD scores of children with PDD would significantly correlate with PDD, ADHD and ODD symptoms at all time periods of the day was partly confirmed, but significantly strong correlations were observed in more areas of the ADHD-RS and ODBI scores compared with the TABS scores. The association of the QCD scores was greater with ADHD-RS and ODBI scores than that with TABS scores. Therefore, the minor hypothesis that the QCD scores of children with PDD would correlate significantly with PDD symptoms at all time periods of the day was rejected.

Although other measures such as the Autism Diagnostic Interview-Revised and the Autism Diagnostic Observation Schedule may alter observed correlations, our results indicate that children with PDD experience difficulties in daily functioning. Particularly, these are associated with both ADHD and ODD symptoms in addition to their diagnosed PDD symptoms. The TABS is a questionnaire for evaluating screening and severity of PDD symptoms. However, Clinicians cannot decide what PDD symptom is a most severe symptom of the child using the TABS. Therefore, this study couldn't indicate that what PDD symptoms may correlate with the lowest QCD for boys and girls of the two age groups. Furthermore, the Diagnostic and Statistical Manual of Mental Disorders, Fifth Edition, allowed diagnosis of ADHD along with comorbid autism spectrum disorder [21]. Therefore, we propose a renewed focus that emphasizes the need to assess patients for potentially comorbid symptoms of ADHD and ODD symptoms at separate time periods of the day before initiating therapy for PDD.

The minor hypothesis that the QCD scores of males with PDD would be significantly lower compared with those of females was partly confirmed. The total QCD scores, “School” and “after school” subscores were significantly lower for males than that for females in the children with PDD for elementary school students. The parents having children with PDD in elementary school expressed more concerns for males than that for females. Furthermore, “School” and “after school” subscores indicate that females could engage in school and after-school activities. The parents having children with PDD in junior high school expressed concerns regardless of gender.

This study has some limitations that must be considered. The PDD diagnosis was made after two or three examinations, and further examinations may have led to changes in diagnoses. In addition, the accuracy of all four measures may be subject to recall bias, subjective reporting, and other types of response errors. Another limitation is the presence of differences between case and control populations. Controls were recruited from the general population of a single district, whereas children with PDD were recruited from a national center in Japan. Although the national center was located in the same district, outpatients at the center belonged to different districts. In addition, controls did not undergo any psychiatric evaluation; therefore, we cannot exclude the presence of mental disorders in these children. The last limitation is that this study compared cases and controls evenly in puberty age. Participants were divided into a PDD group (cases) and a community group (controls) for elementary and junior high school students, respectively. We thought the age difference between the cases (13.3 ± 1.00) and controls (13.1 ± 0.92) for junior high school students (p<0.05 and effect size 0.21) may not influence the results of the QCD, TABS ADHD-RS and ODBI scores in each of the groups differently. But this study did not consider the changes of puberty like adolescent rebellious behavior. Puberty age is a time impulse control is difficult. Parents might evaluate adolescent rebellious behavior as ADHD and ODD symptoms of ADHD-RS and ODBI.

In summary, this study determined that children with PDD experience more difficulties in daily functioning compared with a community sample of children. These difficulties differ for gender and vary over the day. The use of QCD in children with PDD enables clinicians to elucidate problems in their daily life during specific time periods of the day. QCD has three important characteristics: the ability to evaluate life function, the capacity to evaluate behavior throughout the day, and ease of use in daily practice. However, several factors need further consideration before determining reference values and cutoff scores. Finally, care should be taken when generalizing current results to wider populations because future studies including participants from a cross-section of districts are required.

## Supporting Information

S1 Appendix Questionnaire-Children with Difficulties. To prevent misinterpretation and biases, two Japanese psychiatrists with a good command of English, who understood the background and objectives of the evaluation scale, independently carried out forward translation of the QCD into English. Then, the two translators discussed and integrated the two translated versions into one. Another psychiatrist did back translation to Japanese, the original language. The back-translated version was examined by the author of the original version and it was confirmed that the intent of the author was accurately translated. After the final proofreading, construction of the QCD English version was completed.
(PDF)
